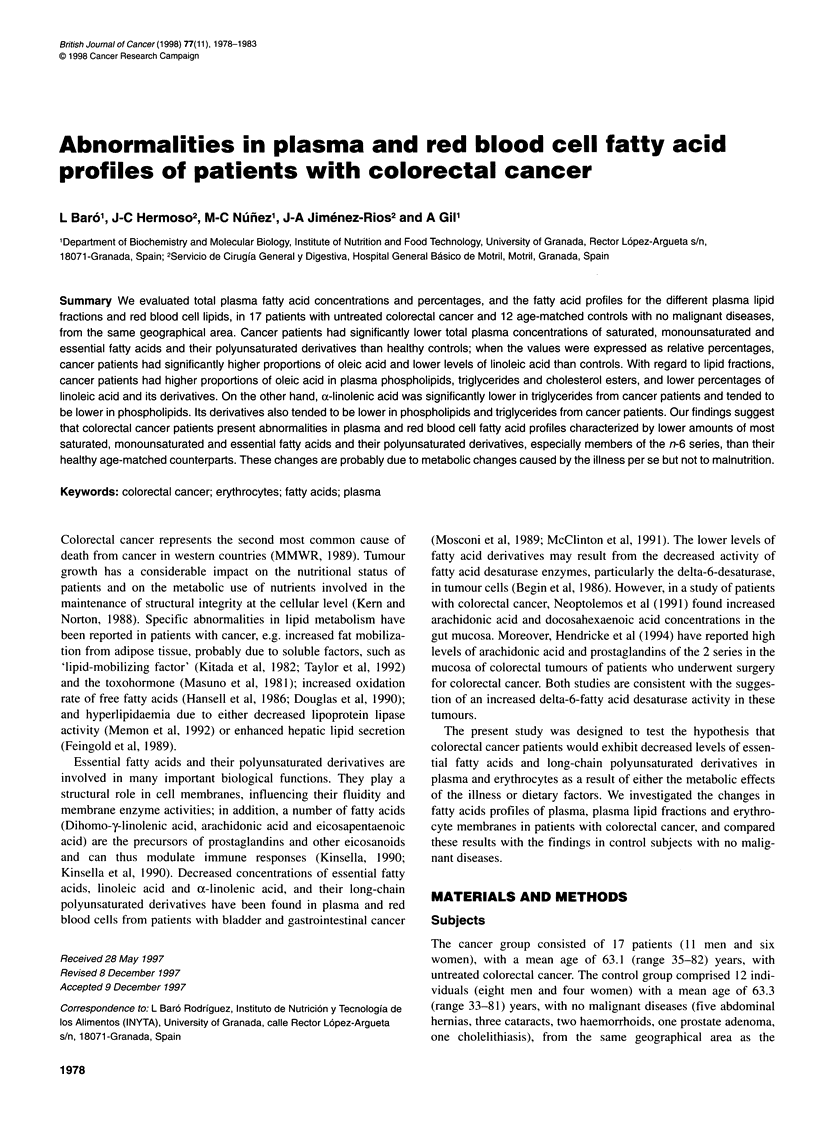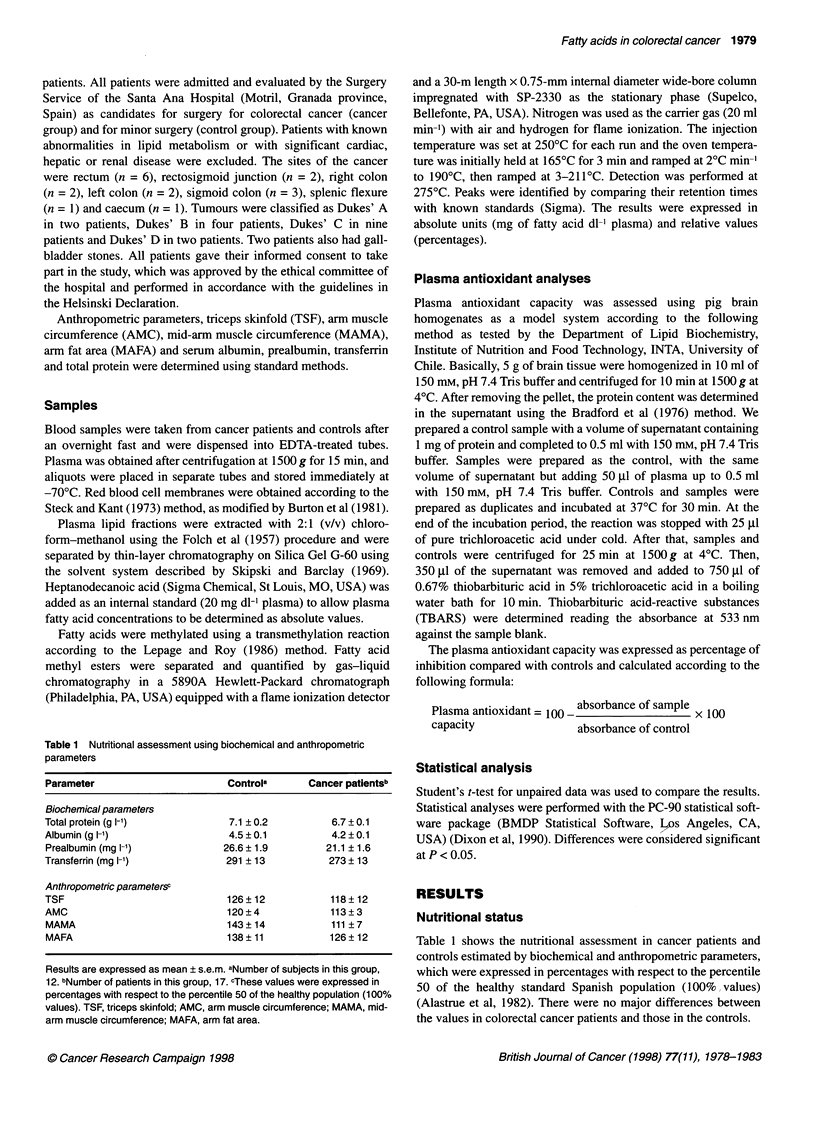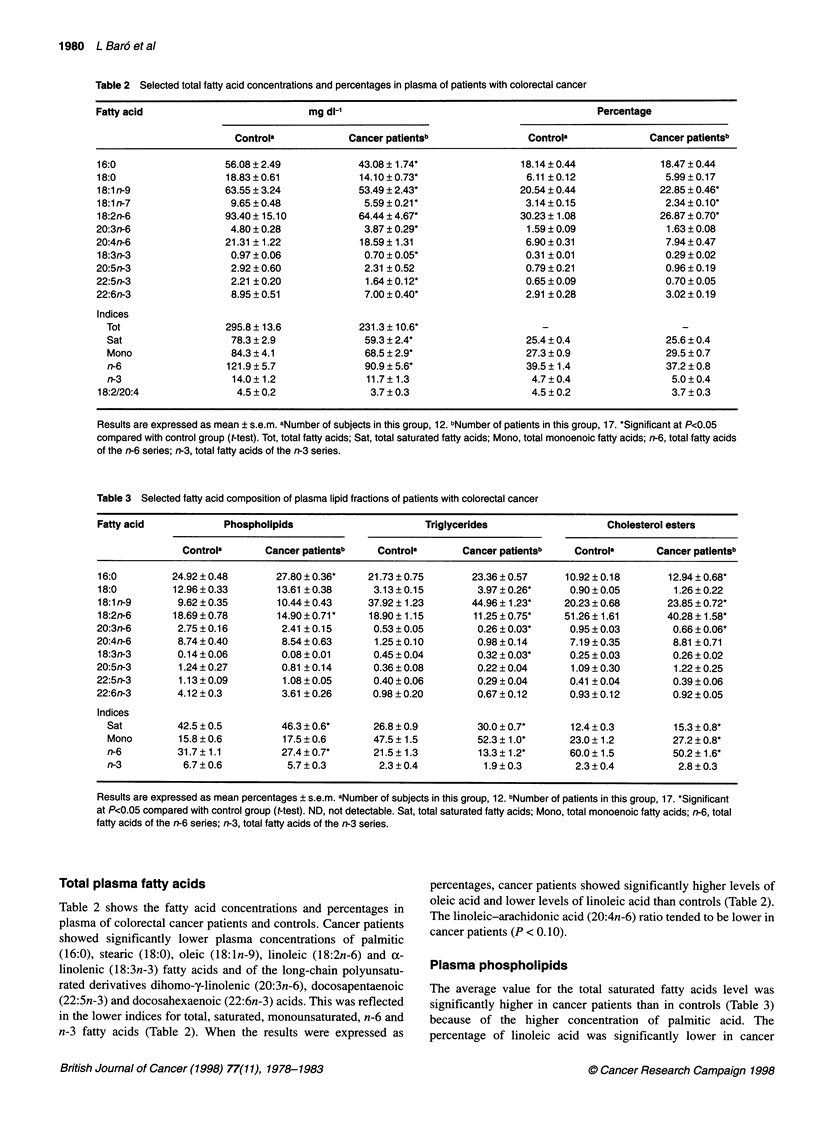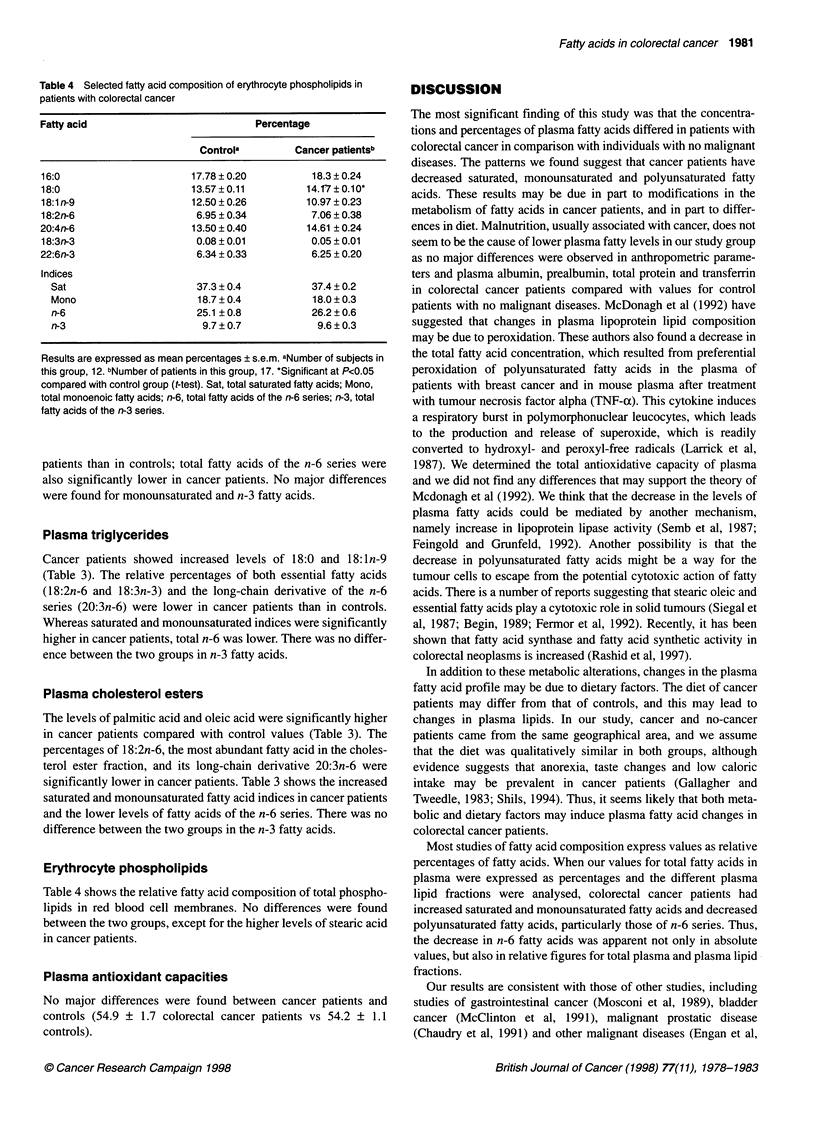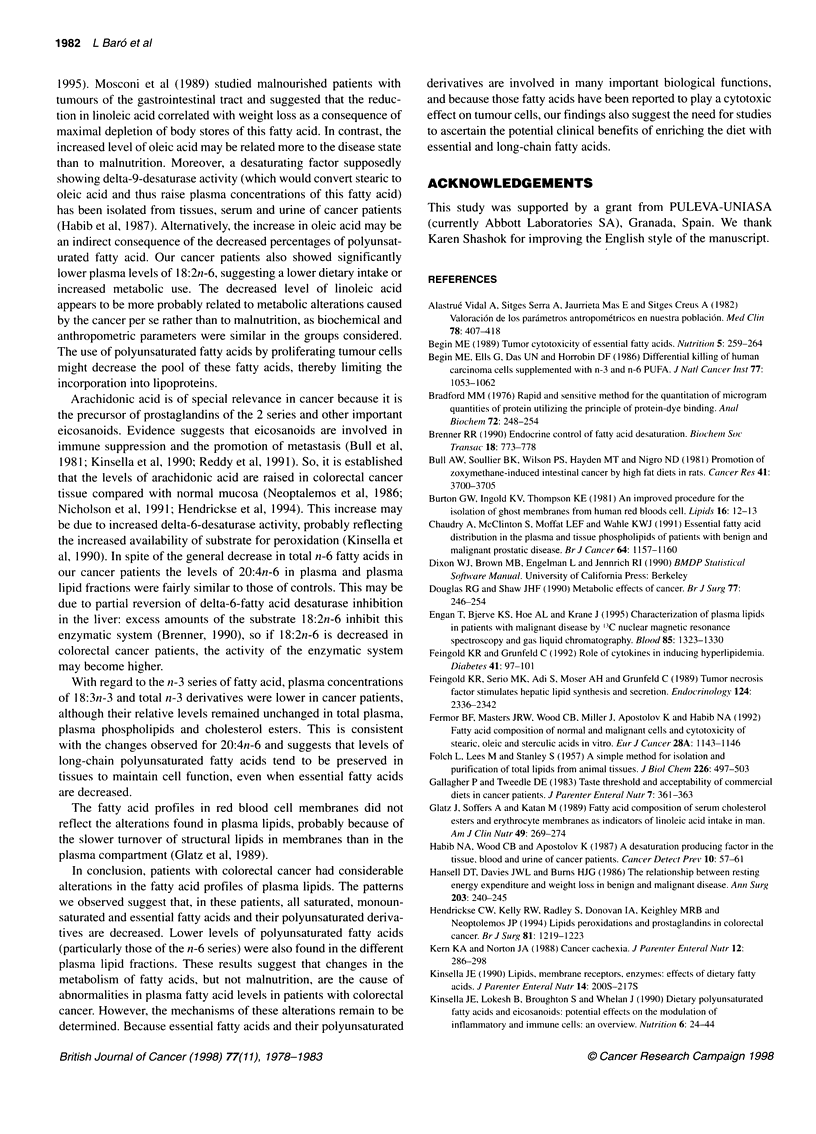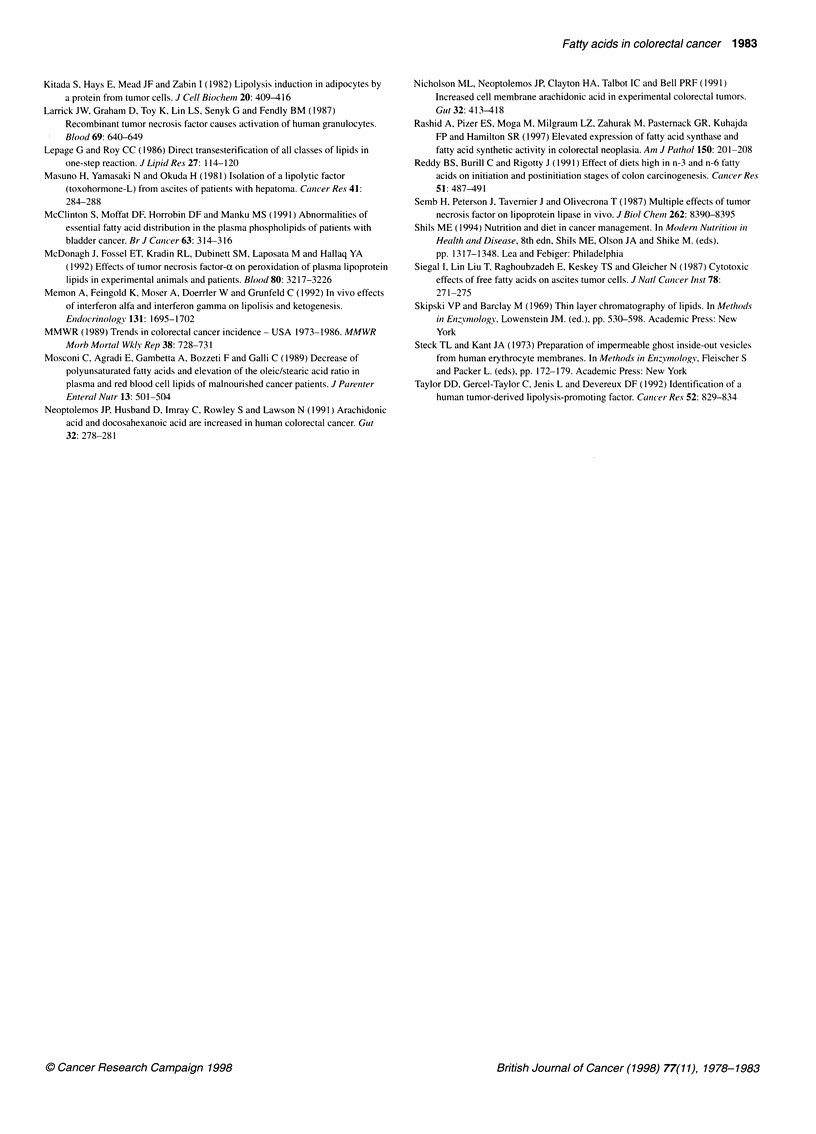# Abnormalities in plasma and red blood cell fatty acid profiles of patients with colorectal cancer.

**DOI:** 10.1038/bjc.1998.328

**Published:** 1998-06

**Authors:** L. BarÃ³, J. C. Hermoso, M. C. NÃºÃ±ez, J. A. JimÃ©nez-Rios, A. Gil

**Affiliations:** Department of Biochemistry and Molecular Biology, Institute of Nutrition and Food Technology, University of Granada, Spain.

## Abstract

We evaluated total plasma fatty acid concentrations and percentages, and the fatty acid profiles for the different plasma lipid fractions and red blood cell lipids, in 17 patients with untreated colorectal cancer and 12 age-matched controls with no malignant diseases, from the same geographical area. Cancer patients had significantly lower total plasma concentrations of saturated, monounsaturated and essential fatty acids and their polyunsaturated derivatives than healthy controls; when the values were expressed as relative percentages, cancer patients had significantly higher proportions of oleic acid and lower levels of linoleic acid than controls. With regard to lipid fractions, cancer patients had higher proportions of oleic acid in plasma phospholipids, triglycerides and cholesterol esters, and lower percentages of linoleic acid and its derivatives. On the other hand, alpha-linolenic acid was significantly lower in triglycerides from cancer patients and tended to be lower in phospholipids. Its derivatives also tended to be lower in phospholipids and triglycerides from cancer patients. Our findings suggest that colorectal cancer patients present abnormalities in plasma and red blood cell fatty acid profiles characterized by lower amounts of most saturated, monounsaturated and essential fatty acids and their polyunsaturated derivatives, especially members of the n-6 series, than their healthy age-matched counterparts. These changes are probably due to metabolic changes caused by the illness per se but not to malnutrition.


British Journal of Cancer (1998) 77(11), 1978–1983
© 1998 Cancer Research Campaign

### Abnormalities in plasma and red blood cell fatty acid profiles of patients with colorectal cancer

**L Baró[1], J-C Hermoso[2], M-C Núñez[1], J-A Jiménez-Rios[2] and A Gil[1]**

[1]Department of Biochemistry and Molecular Biology, Institute of Nutrition and Food Technology, University of Granada, Rector López-Argueta s/n, 18071-Granada, Spain; [2]Servicio de Cirugía General y Digestiva, Hospital General Básico de Motril, Motril, Granada, Spain

**Summary** We evaluated total plasma fatty acid concentrations and percentages, and the fatty acid profiles for the different plasma lipid fractions and red blood cell lipids, in 17 patients with untreated colorectal cancer and 12 age-matched controls with no malignant diseases, from the same geographical area. Cancer patients had significantly lower total plasma concentrations of saturated, monounsaturated and essential fatty acids and their polyunsaturated derivatives than healthy controls; when the values were expressed as relative percentages, cancer patients had significantly higher proportions of oleic acid and lower levels of linoleic acid than controls. With regard to lipid fractions, cancer patients had higher proportions of oleic acid in plasma phospholipids, triglycerides and cholesterol esters, and lower percentages of linoleic acid and its derivatives. On the other hand, α-linolenic acid was significantly lower in triglycerides from cancer patients and tended to be lower in phospholipids. Its derivatives also tended to be lower in phospholipids and triglycerides from cancer patients. Our findings suggest that colorectal cancer patients present abnormalities in plasma and red blood cell fatty acid profiles characterized by lower amounts of most saturated, monounsaturated and essential fatty acids and their polyunsaturated derivatives, especially members of the *n*-6 series, than their healthy age-matched counterparts. These changes are probably due to metabolic changes caused by the illness per se but not to malnutrition.

**Keywords:** colorectal cancer; erythrocytes; fatty acids; plasma

Colorectal cancer represents the second most common cause of death from cancer in western countries (MMWR, 1989). Tumour growth has a considerable impact on the nutritional status of patients and on the metabolic use of nutrients involved in the maintenance of structural integrity at the cellular level (Kern and Norton, 1988). Specific abnormalities in lipid metabolism have been reported in patients with cancer, e.g. increased fat mobilization from adipose tissue, probably due to soluble factors, such as 'lipid-mobilizing factor' (Kitada et al, 1982; Taylor et al, 1992) and the toxohormone (Masuno et al, 1981); increased oxidation rate of free fatty acids (Hansell et al, 1986; Douglas et al, 1990); and hyperlipidaemia due to either decreased lipoprotein lipase activity (Memon et al, 1992) or enhanced hepatic lipid secretion (Feingold et al, 1989).

Essential fatty acids and their polyunsaturated derivatives are involved in many important biological functions. They play a structural role in cell membranes, influencing their fluidity and membrane enzyme activities; in addition, a number of fatty acids (Dihomo-γ-linolenic acid, arachidonic acid and eicosapentaenoic acid) are the precursors of prostaglandins and other eicosanoids and can thus modulate immune responses (Kinsella, 1990; Kinsella et al, 1990). Decreased concentrations of essential fatty acids, linoleic acid and α-linolenic acid, and their long-chain polyunsaturated derivatives have been found in plasma and red blood cells from patients with bladder and gastrointestinal cancer (Mosconi et al, 1989; McClinton et al, 1991). The lower levels of fatty acid derivatives may result from the decreased activity of fatty acid desaturase enzymes, particularly the delta-6-desaturase, in tumour cells (Begin et al, 1986). However, in a study of patients with colorectal cancer, Neoptolemos et al (1991) found increased arachidonic acid and docosahexaenoic acid concentrations in the gut mucosa. Moreover, Hendricke et al (1994) have reported high levels of arachidonic acid and prostaglandins of the 2 series in the mucosa of colorectal tumours of patients who underwent surgery for colorectal cancer. Both studies are consistent with the suggestion of an increased delta-6-fatty acid desaturase activity in these tumours.

The present study was designed to test the hypothesis that colorectal cancer patients would exhibit decreased levels of essential fatty acids and long-chain polyunsaturated derivatives in plasma and erythrocytes as a result of either the metabolic effects of the illness or dietary factors. We investigated the changes in fatty acids profiles of plasma, plasma lipid fractions and erythrocyte membranes in patients with colorectal cancer, and compared these results with the findings in control subjects with no malignant diseases.

### MATERIALS AND METHODS

### Subjects

The cancer group consisted of 17 patients (11 men and six women), with a mean age of 63.1 (range 35–82) years, with untreated colorectal cancer. The control group comprised 12 individuals (eight men and four women) with a mean age of 63.3 (range 33–81) years, with no malignant diseases (five abdominal hernias, three cataracts, two haemorrhoids, one prostate adenoma, one cholelithiasis), from the same geographical area as the

*Received 28 May 1997*
*Revised 8 December 1997*
*Accepted 9 December 1997*

*Correspondence to:* L Baró Rodríguez, Instituto de Nutrición y Tecnología de los Alimentos (INYTA), University of Granada, calle Rector López-Argueta s/n, 18071-Granada, Spain patients. All patients were admitted and evaluated by the Surgery Service of the Santa Ana Hospital (Motril, Granada province, Spain) as candidates for surgery for colorectal cancer (cancer group) and for minor surgery (control group). Patients with known abnormalities in lipid metabolism or with significant cardiac, hepatic or renal disease were excluded. The sites of the cancer were rectum ($n$ = 6), rectosigmoid junction ($n$ = 2), right colon ($n$ = 2), left colon ($n$ = 2), sigmoid colon ($n$ = 3), splenic flexure ($n$ = 1) and caecum ($n$ = 1). Tumours were classified as Dukes' A in two patients, Dukes' B in four patients, Dukes' C in nine patients and Dukes' D in two patients. Two patients also had gall-bladder stones. All patients gave their informed consent to take part in the study, which was approved by the ethical committee of the hospital and performed in accordance with the guidelines in the Helsinski Declaration.

Anthropometric parameters, triceps skinfold (TSF), arm muscle circumference (AMC), mid-arm muscle circumference (MAMA), arm fat area (MAFA) and serum albumin, prealbumin, transferrin and total protein were determined using standard methods.

### Samples

Blood samples were taken from cancer patients and controls after an overnight fast and were dispensed into EDTA-treated tubes. Plasma was obtained after centrifugation at 1500 $g$ for 15 min, and aliquots were placed in separate tubes and stored immediately at –70°C. Red blood cell membranes were obtained according to the Steck and Kant (1973) method, as modified by Burton et al (1981).

Plasma lipid fractions were extracted with 2:1 (v/v) chloroform–methanol using the Folch et al (1957) procedure and were separated by thin-layer chromatography on Silica Gel G-60 using the solvent system described by Skipski and Barclay (1969). Heptanodecanoic acid (Sigma Chemical, St Louis, MO, USA) was added as an internal standard (20 mg dl$^{-1}$ plasma) to allow plasma fatty acid concentrations to be determined as absolute values.

Fatty acids were methylated using a transmethylation reaction according to the Lepage and Roy (1986) method. Fatty acid methyl esters were separated and quantified by gas–liquid chromatography in a 5890A Hewlett-Packard chromatograph (Philadelphia, PA, USA) equipped with a flame ionization detector and a 30-m length × 0.75-mm internal diameter wide-bore column impregnated with SP-2330 as the stationary phase (Supelco, Bellefonte, PA, USA). Nitrogen was used as the carrier gas (20 ml min$^{-1}$) with air and hydrogen for flame ionization. The injection temperature was set at 250°C for each run and the oven temperature was initially held at 165°C for 3 min and ramped at 2°C min$^{-1}$ to 190°C, then ramped at 3–211°C. Detection was performed at 275°C. Peaks were identified by comparing their retention times with known standards (Sigma). The results were expressed in absolute units (mg of fatty acid dl$^{-1}$ plasma) and relative values (percentages).

**Table 1** Nutritional assessment using biochemical and anthropometric parameters

| Parameter | Control[a] | Cancer patients[b] |
|---|---|---|
| *Biochemical parameters* | | |
| Total protein (g l$^{-1}$) | 7.1 ± 0.2 | 6.7 ± 0.1 |
| Albumin (g l$^{-1}$) | 4.5 ± 0.1 | 4.2 ± 0.1 |
| Prealbumin (mg l$^{-1}$) | 26.6 ± 1.9 | 21.1 ± 1.6 |
| Transferrin (mg l$^{-1}$) | 291 ± 13 | 273 ± 13 |
| *Anthropometric parameters*[c] | | |
| TSF | 126 ± 12 | 118 ± 12 |
| AMC | 120 ± 4 | 113 ± 3 |
| MAMA | 143 ± 14 | 111 ± 7 |
| MAFA | 138 ± 11 | 126 ± 12 |

Results are expressed as mean ± s.e.m. [a]Number of subjects in this group, 12. [b]Number of patients in this group, 17. [c]These values were expressed in percentages with respect to the percentile 50 of the healthy population (100% values). TSF, triceps skinfold; AMC, arm muscle circumference; MAMA, mid-arm muscle circumference; MAFA, arm fat area.

### Plasma antioxidant analyses

Plasma antioxidant capacity was assessed using pig brain homogenates as a model system according to the following method as tested by the Department of Lipid Biochemistry, Institute of Nutrition and Food Technology, INTA, University of Chile. Basically, 5 g of brain tissue were homogenized in 10 ml of 150 mM, pH 7.4 Tris buffer and centrifuged for 10 min at 1500 $g$ at 4°C. After removing the pellet, the protein content was determined in the supernatant using the Bradford et al (1976) method. We prepared a control sample with a volume of supernatant containing 1 mg of protein and completed to 0.5 ml with 150 mM, pH 7.4 Tris buffer. Samples were prepared as the control, with the same volume of supernatant but adding 50 µl of plasma up to 0.5 ml with 150 mM, pH 7.4 Tris buffer. Controls and samples were prepared as duplicates and incubated at 37°C for 30 min. At the end of the incubation period, the reaction was stopped with 25 µl of pure trichloroacetic acid under cold. After that, samples and controls were centrifuged for 25 min at 1500 $g$ at 4°C. Then, 350 µl of the supernatant was removed and added to 750 µl of 0.67% thiobarbituric acid in 5% trichloroacetic acid in a boiling water bath for 10 min. Thiobarbituric acid-reactive substances (TBARS) were determined reading the absorbance at 533 nm against the sample blank.

The plasma antioxidant capacity was expressed as percentage of inhibition compared with controls and calculated according to the following formula:

$$\text{Plasma antioxidant capacity} = 100 - \frac{\text{absorbance of sample}}{\text{absorbance of control}} \times 100$$

### Statistical analysis

Student's $t$-test for unpaired data was used to compare the results. Statistical analyses were performed with the PC-90 statistical software package (BMDP Statistical Software, Los Angeles, CA, USA) (Dixon et al, 1990). Differences were considered significant at $P < 0.05$.

### RESULTS

### Nutritional status

Table 1 shows the nutritional assessment in cancer patients and controls estimated by biochemical and anthropometric parameters, which were expressed in percentages with respect to the percentile 50 of the healthy standard Spanish population (100% values) (Alastrue et al, 1982). There were no major differences between the values in colorectal cancer patients and those in the controls.

**Table 2** Selected total fatty acid concentrations and percentages in plasma of patients with colorectal cancer

| Fatty acid | mg dl$^{-1}$ | | Percentage | |
|---|---|---|---|---|
| | Control[a] | Cancer patients[b] | Control[a] | Cancer patients[b] |
| 16:0 | 56.08 ± 2.49 | 43.08 ± 1.74* | 18.14 ± 0.44 | 18.47 ± 0.44 |
| 18:0 | 18.83 ± 0.61 | 14.10 ± 0.73* | 6.11 ± 0.12 | 5.99 ± 0.17 |
| 18:1*n*-9 | 63.55 ± 3.24 | 53.49 ± 2.43* | 20.54 ± 0.44 | 22.85 ± 0.46* |
| 18:1*n*-7 | 9.65 ± 0.48 | 5.59 ± 0.21* | 3.14 ± 0.15 | 2.34 ± 0.10* |
| 18:2*n*-6 | 93.40 ± 15.10 | 64.44 ± 4.67* | 30.23 ± 1.08 | 26.87 ± 0.70* |
| 20:3*n*-6 | 4.80 ± 0.28 | 3.87 ± 0.29* | 1.59 ± 0.09 | 1.63 ± 0.08 |
| 20:4*n*-6 | 21.31 ± 1.22 | 18.59 ± 1.31 | 6.90 ± 0.31 | 7.94 ± 0.47 |
| 18:3*n*-3 | 0.97 ± 0.06 | 0.70 ± 0.05* | 0.31 ± 0.01 | 0.29 ± 0.02 |
| 20:5*n*-3 | 2.92 ± 0.60 | 2.31 ± 0.52 | 0.79 ± 0.21 | 0.96 ± 0.19 |
| 22:5*n*-3 | 2.21 ± 0.20 | 1.64 ± 0.12* | 0.65 ± 0.09 | 0.70 ± 0.05 |
| 22:6*n*-3 | 8.95 ± 0.51 | 7.00 ± 0.40* | 2.91 ± 0.28 | 3.02 ± 0.19 |
| Indices | | | | |
| Tot | 295.8 ± 13.6 | 231.3 ± 10.6* | – | – |
| Sat | 78.3 ± 2.9 | 59.3 ± 2.4* | 25.4 ± 0.4 | 25.6 ± 0.4 |
| Mono | 84.3 ± 4.1 | 68.5 ± 2.9* | 27.3 ± 0.9 | 29.5 ± 0.7 |
| *n*-6 | 121.9 ± 5.7 | 90.9 ± 5.6* | 39.5 ± 1.4 | 37.2 ± 0.8 |
| *n*-3 | 14.0 ± 1.2 | 11.7 ± 1.3 | 4.7 ± 0.4 | 5.0 ± 0.4 |
| 18:2/20:4 | 4.5 ± 0.2 | 3.7 ± 0.3 | 4.5 ± 0.2 | 3.7 ± 0.3 |

Results are expressed as mean ± s.e.m. [a]Number of subjects in this group, 12. [b]Number of patients in this group, 17. *Significant at $P<0.05$ compared with control group (*t*-test). Tot, total fatty acids; Sat, total saturated fatty acids; Mono, total monoenoic fatty acids; *n*-6, total fatty acids of the *n*-6 series; *n*-3, total fatty acids of the *n*-3 series.

**Table 3** Selected fatty acid composition of plasma lipid fractions of patients with colorectal cancer

| Fatty acid | Phospholipids | | Triglycerides | | Cholesterol esters | |
|---|---|---|---|---|---|---|
| | Control[a] | Cancer patients[b] | Control[a] | Cancer patients[b] | Control[a] | Cancer patients[b] |
| 16:0 | 24.92 ± 0.48 | 27.80 ± 0.36* | 21.73 ± 0.75 | 23.36 ± 0.57 | 10.92 ± 0.18 | 12.94 ± 0.68* |
| 18:0 | 12.96 ± 0.33 | 13.61 ± 0.38 | 3.13 ± 0.15 | 3.97 ± 0.26* | 0.90 ± 0.05 | 1.26 ± 0.22 |
| 18:1*n*-9 | 9.62 ± 0.35 | 10.44 ± 0.43 | 37.92 ± 1.23 | 44.96 ± 1.23* | 20.23 ± 0.68 | 23.85 ± 0.72* |
| 18:2*n*-6 | 18.69 ± 0.78 | 14.90 ± 0.71* | 18.90 ± 1.15 | 11.25 ± 0.75* | 51.26 ± 1.61 | 40.28 ± 1.58* |
| 20:3*n*-6 | 2.75 ± 0.16 | 2.41 ± 0.15 | 0.53 ± 0.05 | 0.26 ± 0.03* | 0.95 ± 0.03 | 0.66 ± 0.06* |
| 20:4*n*-6 | 8.74 ± 0.40 | 8.54 ± 0.63 | 1.25 ± 0.10 | 0.98 ± 0.14 | 7.19 ± 0.35 | 8.81 ± 0.71 |
| 18:3*n*-3 | 0.14 ± 0.06 | 0.08 ± 0.01 | 0.45 ± 0.04 | 0.32 ± 0.03* | 0.25 ± 0.03 | 0.26 ± 0.02 |
| 20:5*n*-3 | 1.24 ± 0.27 | 0.81 ± 0.14 | 0.36 ± 0.08 | 0.22 ± 0.04 | 1.09 ± 0.30 | 1.22 ± 0.25 |
| 22:5*n*-3 | 1.13 ± 0.09 | 1.08 ± 0.05 | 0.40 ± 0.06 | 0.29 ± 0.04 | 0.41 ± 0.04 | 0.39 ± 0.06 |
| 22:6*n*-3 | 4.12 ± 0.3 | 3.61 ± 0.26 | 0.98 ± 0.20 | 0.67 ± 0.12 | 0.93 ± 0.12 | 0.92 ± 0.05 |
| Indices | | | | | | |
| Sat | 42.5 ± 0.5 | 46.3 ± 0.6* | 26.8 ± 0.9 | 30.0 ± 0.7* | 12.4 ± 0.3 | 15.3 ± 0.8* |
| Mono | 15.8 ± 0.6 | 17.5 ± 0.6 | 47.5 ± 1.5 | 52.3 ± 1.0* | 23.0 ± 1.2 | 27.2 ± 0.8* |
| *n*-6 | 31.7 ± 1.1 | 27.4 ± 0.7* | 21.5 ± 1.3 | 13.3 ± 1.2* | 60.0 ± 1.5 | 50.2 ± 1.6* |
| *n*-3 | 6.7 ± 0.6 | 5.7 ± 0.3 | 2.3 ± 0.4 | 1.9 ± 0.3 | 2.3 ± 0.4 | 2.8 ± 0.3 |

Results are expressed as mean percentages ± s.e.m. [a]Number of subjects in this group, 12. [b]Number of patients in this group, 17. *Significant at $P<0.05$ compared with control group (*t*-test). ND, not detectable. Sat, total saturated fatty acids; Mono, total monoenoic fatty acids; *n*-6, total fatty acids of the *n*-6 series; *n*-3, total fatty acids of the *n*-3 series.

### Total plasma fatty acids

Table 2 shows the fatty acid concentrations and percentages in plasma of colorectal cancer patients and controls. Cancer patients showed significantly lower plasma concentrations of palmitic (16:0), stearic (18:0), oleic (18:1*n*-9), linoleic (18:2*n*-6) and α-linolenic (18:3*n*-3) fatty acids and of the long-chain polyunsaturated derivatives dihomo-γ-linolenic (20:3*n*-6), docosapentaenoic (22:5*n*-3) and docosahexaenoic (22:6*n*-3) acids. This was reflected in the lower indices for total, saturated, monounsaturated, *n*-6 and *n*-3 fatty acids (Table 2). When the results were expressed as percentages, cancer patients showed significantly higher levels of oleic acid and lower levels of linoleic acid than controls (Table 2). The linoleic–arachidonic acid (20:4*n*-6) ratio tended to be lower in cancer patients ($P < 0.10$).

### Plasma phospholipids

The average value for the total saturated fatty acids level was significantly higher in cancer patients than in controls (Table 3) because of the higher concentration of palmitic acid. The percentage of linoleic acid was significantly lower in cancer

 © Cancer Research Campaign 1998

**Table 4** Selected fatty acid composition of erythrocyte phospholipids in patients with colorectal cancer

| Fatty acid | Percentage | |
|---|---|---|
| | Control[a] | Cancer patients[b] |
| 16:0 | 17.78 ± 0.20 | 18.3 ± 0.24 |
| 18:0 | 13.57 ± 0.11 | 14.17 ± 0.10* |
| 18:1*n*-9 | 12.50 ± 0.26 | 10.97 ± 0.23 |
| 18:2*n*-6 | 6.95 ± 0.34 | 7.06 ± 0.38 |
| 20:4*n*-6 | 13.50 ± 0.40 | 14.61 ± 0.24 |
| 18:3*n*-3 | 0.08 ± 0.01 | 0.05 ± 0.01 |
| 22:6*n*-3 | 6.34 ± 0.33 | 6.25 ± 0.20 |
| Indices | | |
| Sat | 37.3 ± 0.4 | 37.4 ± 0.2 |
| Mono | 18.7 ± 0.4 | 18.0 ± 0.3 |
| *n*-6 | 25.1 ± 0.8 | 26.2 ± 0.6 |
| *n*-3 | 9.7 ± 0.7 | 9.6 ± 0.3 |

Results are expressed as mean percentages ± s.e.m. [a]Number of subjects in this group, 12. [b]Number of patients in this group, 17. *Significant at $P<0.05$ compared with control group (*t*-test). Sat, total saturated fatty acids; Mono, total monoenoic fatty acids; *n*-6, total fatty acids of the *n*-6 series; *n*-3, total fatty acids of the *n*-3 series.

patients than in controls; total fatty acids of the *n*-6 series were also significantly lower in cancer patients. No major differences were found for monounsaturated and *n*-3 fatty acids.

### Plasma triglycerides

Cancer patients showed increased levels of 18:0 and 18:1*n*-9 (Table 3). The relative percentages of both essential fatty acids (18:2*n*-6 and 18:3*n*-3) and the long-chain derivative of the *n*-6 series (20:3*n*-6) were lower in cancer patients than in controls. Whereas saturated and monounsaturated indices were significantly higher in cancer patients, total *n*-6 was lower. There was no difference between the two groups in *n*-3 fatty acids.

### Plasma cholesterol esters

The levels of palmitic acid and oleic acid were significantly higher in cancer patients compared with control values (Table 3). The percentages of 18:2*n*-6, the most abundant fatty acid in the cholesterol ester fraction, and its long-chain derivative 20:3*n*-6 were significantly lower in cancer patients. Table 3 shows the increased saturated and monounsaturated fatty acid indices in cancer patients and the lower levels of fatty acids of the *n*-6 series. There was no difference between the two groups in the *n*-3 fatty acids.

### Erythrocyte phospholipids

Table 4 shows the relative fatty acid composition of total phospholipids in red blood cell membranes. No differences were found between the two groups, except for the higher levels of stearic acid in cancer patients.

### Plasma antioxidant capacities

No major differences were found between cancer patients and controls (54.9 ± 1.7 colorectal cancer patients vs 54.2 ± 1.1 controls).

### DISCUSSION

The most significant finding of this study was that the concentrations and percentages of plasma fatty acids differed in patients with colorectal cancer in comparison with individuals with no malignant diseases. The patterns we found suggest that cancer patients have decreased saturated, monounsaturated and polyunsaturated fatty acids. These results may be due in part to modifications in the metabolism of fatty acids in cancer patients, and in part to differences in diet. Malnutrition, usually associated with cancer, does not seem to be the cause of lower plasma fatty levels in our study group as no major differences were observed in anthropometric parameters and plasma albumin, prealbumin, total protein and transferrin in colorectal cancer patients compared with values for control patients with no malignant diseases. McDonagh et al (1992) have suggested that changes in plasma lipoprotein lipid composition may be due to peroxidation. These authors also found a decrease in the total fatty acid concentration, which resulted from preferential peroxidation of polyunsaturated fatty acids in the plasma of patients with breast cancer and in mouse plasma after treatment with tumour necrosis factor alpha (TNF-α). This cytokine induces a respiratory burst in polymorphonuclear leucocytes, which leads to the production and release of superoxide, which is readily converted to hydroxyl- and peroxyl-free radicals (Larrick et al, 1987). We determined the total antioxidative capacity of plasma and we did not find any differences that may support the theory of Mcdonagh et al (1992). We think that the decrease in the levels of plasma fatty acids could be mediated by another mechanism, namely increase in lipoprotein lipase activity (Semb et al, 1987; Feingold and Grunfeld, 1992). Another possibility is that the decrease in polyunsaturated fatty acids might be a way for the tumour cells to escape from the potential cytotoxic action of fatty acids. There is a number of reports suggesting that stearic oleic and essential fatty acids play a cytotoxic role in solid tumours (Siegal et al, 1987; Begin, 1989; Fermor et al, 1992). Recently, it has been shown that fatty acid synthase and fatty acid synthetic activity in colorectal neoplasms is increased (Rashid et al, 1997).

In addition to these metabolic alterations, changes in the plasma fatty acid profile may be due to dietary factors. The diet of cancer patients may differ from that of controls, and this may lead to changes in plasma lipids. In our study, cancer and no-cancer patients came from the same geographical area, and we assume that the diet was qualitatively similar in both groups, although evidence suggests that anorexia, taste changes and low caloric intake may be prevalent in cancer patients (Gallagher and Tweedle, 1983; Shils, 1994). Thus, it seems likely that both metabolic and dietary factors may induce plasma fatty acid changes in colorectal cancer patients.

Most studies of fatty acid composition express values as relative percentages of fatty acids. When our values for total fatty acids in plasma were expressed as percentages and the different plasma lipid fractions were analysed, colorectal cancer patients had increased saturated and monounsaturated fatty acids and decreased polyunsaturated fatty acids, particularly those of *n*-6 series. Thus, the decrease in *n*-6 fatty acids was apparent not only in absolute values, but also in relative figures for total plasma and plasma lipid fractions.

Our results are consistent with those of other studies, including studies of gastrointestinal cancer (Mosconi et al, 1989), bladder cancer (McClinton et al, 1991), malignant prostatic disease (Chaudry et al, 1991) and other malignant diseases (Engan et al,

© Cancer Research Campaign 1998

1995). Mosconi et al (1989) studied malnourished patients with tumours of the gastrointestinal tract and suggested that the reduction in linoleic acid correlated with weight loss as a consequence of maximal depletion of body stores of this fatty acid. In contrast, the increased level of oleic acid may be related more to the disease state than to malnutrition. Moreover, a desaturating factor supposedly showing delta-9-desaturase activity (which would convert stearic to oleic acid and thus raise plasma concentrations of this fatty acid) has been isolated from tissues, serum and urine of cancer patients (Habib et al, 1987). Alternatively, the increase in oleic acid may be an indirect consequence of the decreased percentages of polyunsaturated fatty acid. Our cancer patients also showed significantly lower plasma levels of 18:2*n*-6, suggesting a lower dietary intake or increased metabolic use. The decreased level of linoleic acid appears to be more probably related to metabolic alterations caused by the cancer per se rather than to malnutrition, as biochemical and anthropometric parameters were similar in the groups considered. The use of polyunsaturated fatty acids by proliferating tumour cells might decrease the pool of these fatty acids, thereby limiting the incorporation into lipoproteins.

Arachidonic acid is of special relevance in cancer because it is the precursor of prostaglandins of the 2 series and other important eicosanoids. Evidence suggests that eicosanoids are involved in immune suppression and the promotion of metastasis (Bull et al, 1981; Kinsella et al, 1990; Reddy et al, 1991). So, it is established that the levels of arachidonic acid are raised in colorectal cancer tissue compared with normal mucosa (Neoptalemos et al, 1986; Nicholson et al, 1991; Hendrickse et al, 1994). This increase may be due to increased delta-6-desaturase activity, probably reflecting the increased availability of substrate for peroxidation (Kinsella et al, 1990). In spite of the general decrease in total *n*-6 fatty acids in our cancer patients the levels of 20:4*n*-6 in plasma and plasma lipid fractions were fairly similar to those of controls. This may be due to partial reversion of delta-6-fatty acid desaturase inhibition in the liver: excess amounts of the substrate 18:2*n*-6 inhibit this enzymatic system (Brenner, 1990), so if 18:2*n*-6 is decreased in colorectal cancer patients, the activity of the enzymatic system may become higher.

With regard to the *n*-3 series of fatty acid, plasma concentrations of 18:3*n*-3 and total *n*-3 derivatives were lower in cancer patients, although their relative levels remained unchanged in total plasma, plasma phospholipids and cholesterol esters. This is consistent with the changes observed for 20:4*n*-6 and suggests that levels of long-chain polyunsaturated fatty acids tend to be preserved in tissues to maintain cell function, even when essential fatty acids are decreased.

The fatty acid profiles in red blood cell membranes did not reflect the alterations found in plasma lipids, probably because of the slower turnover of structural lipids in membranes than in the plasma compartment (Glatz et al, 1989).

In conclusion, patients with colorectal cancer had considerable alterations in the fatty acid profiles of plasma lipids. The patterns we observed suggest that, in these patients, all saturated, monounsaturated and essential fatty acids and their polyunsaturated derivatives are decreased. Lower levels of polyunsaturated fatty acids (particularly those of the *n*-6 series) were also found in the different plasma lipid fractions. These results suggest that changes in the metabolism of fatty acids, but not malnutrition, are the cause of abnormalities in plasma fatty acid levels in patients with colorectal cancer. However, the mechanisms of these alterations remain to be determined. Because essential fatty acids and their polyunsaturated derivatives are involved in many important biological functions, and because those fatty acids have been reported to play a cytotoxic effect on tumour cells, our findings also suggest the need for studies to ascertain the potential clinical benefits of enriching the diet with essential and long-chain fatty acids.

### ACKNOWLEDGEMENTS

This study was supported by a grant from PULEVA-UNIASA (currently Abbott Laboratories SA), Granada, Spain. We thank Karen Shashok for improving the English style of the manuscript.

### REFERENCES

Alastrué Vidal A, Sitges Serra A, Jaurrieta Mas E and Sitges Creus A (1982) Valoración de los parámetros antropométricos en nuestra población. *Med Clin* **78**: 407–418

Begin ME (1989) Tumor cytotoxicity of essential fatty acids. *Nutrition* **5**: 259–264

Begin ME, Ells G, Das UN and Horrobin DF (1986) Differential killing of human carcinoma cells supplemented with n-3 and n-6 PUFA. *J Natl Cancer Inst* **77**: 1053–1062

Bradford MM (1976) Rapid and sensitive method for the quantitation of microgram quantities of protein utilizing the principle of protein-dye binding. *Anal Biochem* **72**: 248–254

Brenner RR (1990) Endocrine control of fatty acid desaturation. *Biochem Soc Transac* **18**: 773–778

Bull AW, Soullier BK, Wilson PS, Hayden MT and Nigro ND (1981) Promotion of zoxymethane-induced intestinal cancer by high fat diets in rats. *Cancer Res* **41**: 3700–3705

Burton GW, Ingold KV, Thompson KE (1981) An improved procedure for the isolation of ghost membranes from human red bloods cell. *Lipids* **16**: 12–13

Chaudry A, McClinton S, Moffat LEF and Wahle KWJ (1991) Essential fatty acid distribution in the plasma and tissue phospholipids of patients with benign and malignant prostatic disease. *Br J Cancer* **64**: 1157–1160

Dixon WJ, Brown MB, Engelman L and Jennrich RI (1990) *BMDP Statistical Software Manual*. University of California Press: Berkeley

Douglas RG and Shaw JHF (1990) Metabolic effects of cancer. *Br J Surg* **77**: 246–254

Engan T, Bjerve KS, Hoe AL and Krane J (1995) Characterization of plasma lipids in patients with malignant disease by $^{13}$C nuclear magnetic resonance spectroscopy and gas liquid chromatography. *Blood* **85**: 1323–1330

Feingold KR and Grunfeld C (1992) Role of cytokines in inducing hyperlipidemia. *Diabetes* **41**: 97–101

Feingold KR, Serio MK, Adi S, Moser AH and Grunfeld C (1989) Tumor necrosis factor stimulates hepatic lipid synthesis and secretion. *Endocrinology* **124**: 2336–2342

Fermor BF, Masters JRW, Wood CB, Miller J, Apostolov K and Habib NA (1992) Fatty acid composition of normal and malignant cells and cytotoxicity of stearic, oleic and sterculic acids in vitro. *Eur J Cancer* **28A**: 1143–1146

Folch L, Lees M and Stanley S (1957) A simple method for isolation and purification of total lipids from animal tissues. *J Biol Chem* **226**: 497–503

Gallagher P and Tweedle DE (1983) Taste threshold and acceptability of commercial diets in cancer patients. *J Parenter Enteral Nutr* **7**: 361–363

Glatz J, Soffers A and Katan M (1989) Fatty acid composition of serum cholesterol esters and erythrocyte membranes as indicators of linoleic acid intake in man. *Am J Clin Nutr* **49**: 269–274

Habib NA, Wood CB and Apostolov K (1987) A desaturation producing factor in the tissue, blood and urine of cancer patients. *Cancer Detect Prev* **10**: 57–61

Hansell DT, Davies JWL and Burns HJG (1986) The relationship between resting energy expenditure and weight loss in benign and malignant disease. *Ann Surg* **203**: 240–245

Hendrickse CW, Kelly RW, Radley S, Donovan IA, Keighley MRB and Neoptolemos JP (1994) Lipids peroxidations and prostaglandins in colorectal cancer. *Br J Surg* **81**: 1219–1223

Kern KA and Norton JA (1988) Cancer cachexia. *J Parenter Enteral Nutr* **12**: 286–298

Kinsella JE (1990) Lipids, membrane receptors, enzymes: effects of dietary fatty acids. *J Parenter Enteral Nutr* **14**: 200S–217S

Kinsella JE, Lokesh B, Broughton S and Whelan J (1990) Dietary polyunsaturated fatty acids and eicosanoids: potential effects on the modulation of inflammatory and immune cells: an overview. *Nutrition* **6**: 24–44

© Cancer Research Campaign 1998

Kitada S, Hays E, Mead JF and Zabin I (1982) Lipolysis induction in adipocytes by a protein from tumor cells. *J Cell Biochem* **20**: 409–416

Larrick JW, Graham D, Toy K, Lin LS, Senyk G and Fendly BM (1987) Recombinant tumor necrosis factor causes activation of human granulocytes. *Blood* **69**: 640–649

Lepage G and Roy CC (1986) Direct transesterification of all classes of lipids in one-step reaction. *J Lipid Res* **27**: 114–120

Masuno H, Yamasaki N and Okuda H (1981) Isolation of a lipolytic factor (toxohormone-L) from ascites of patients with hepatoma. *Cancer Res* **41**: 284–288

McClinton S, Moffat DF, Horrobin DF and Manku MS (1991) Abnormalities of essential fatty acid distribution in the plasma phospholipids of patients with bladder cancer. *Br J Cancer* **63**: 314–316

McDonagh J, Fossel ET, Kradin RL, Dubinett SM, Laposata M and Hallaq YA (1992) Effects of tumor necrosis factor-α on peroxidation of plasma lipoprotein lipids in experimental animals and patients. *Blood* **80**: 3217–3226

Memon A, Feingold K, Moser A, Doerrler W and Grunfeld C (1992) In vivo effects of interferon alfa and interferon gamma on lipolisis and ketogenesis. *Endocrinology* **131**: 1695–1702

MMWR (1989) Trends in colorectal cancer incidence – USA 1973–1986. *MMWR Morb Mortal Wkly Rep* **38**: 728–731

Mosconi C, Agradi E, Gambetta A, Bozzeti F and Galli C (1989) Decrease of polyunsaturated fatty acids and elevation of the oleic/stearic acid ratio in plasma and red blood cell lipids of malnourished cancer patients. *J Parenter Enteral Nutr* **13**: 501–504

Neoptolemos JP, Husband D, Imray C, Rowley S and Lawson N (1991) Arachidonic acid and docosahexanoic acid are increased in human colorectal cancer. *Gut* **32**: 278–281

Nicholson ML, Neoptolemos JP, Clayton HA, Talbot IC and Bell PRF (1991) Increased cell membrane arachidonic acid in experimental colorectal tumors. *Gut* **32**: 413–418

Rashid A, Pizer ES, Moga M, Milgraum LZ, Zahurak M, Pasternack GR, Kuhajda FP and Hamilton SR (1997) Elevated expression of fatty acid synthase and fatty acid synthetic activity in colorectal neoplasia. *Am J Pathol* **150**: 201–208

Reddy BS, Burill C and Rigotty J (1991) Effect of diets high in n-3 and n-6 fatty acids on initiation and postinitiation stages of colon carcinogenesis. *Cancer Res* **51**: 487–491

Semb H, Peterson J, Tavernier J and Olivecrona T (1987) Multiple effects of tumor necrosis factor on lipoprotein lipase in vivo. *J Biol Chem* **262**: 8390–8395

Shils ME (1994) Nutrition and diet in cancer management. In *Modern Nutrition in Health and Disease*, 8th edn, Shils ME, Olson JA and Shike M. (eds), pp. 1317–1348. Lea and Febiger: Philadelphia

Siegal I, Lin Liu T, Raghoubzadeh E, Keskey TS and Gleicher N (1987) Cytotoxic effects of free fatty acids on ascites tumor cells. *J Natl Cancer Inst* **78**: 271–275

Skipski VP and Barclay M (1969) Thin layer chromatography of lipids. In *Methods in Enzymology*, Lowenstein JM. (ed.), pp. 530–598. Academic Press: New York

Steck TL and Kant JA (1973) Preparation of impermeable ghost inside-out vesicles from human erythrocyte membranes. In *Methods in Enzymology*, Fleischer S and Packer L. (eds), pp. 172–179. Academic Press: New York

Taylor DD, Gercel-Taylor C, Jenis L and Devereux DF (1992) Identification of a human tumor-derived lipolysis-promoting factor. *Cancer Res* **52**: 829–834

© Cancer Research Campaign 1998